# SYN1 Mutation Causes X-Linked Toothbrushing Epilepsy in a Chinese Family

**DOI:** 10.3389/fneur.2021.736977

**Published:** 2021-09-20

**Authors:** Qin Zhou, Jingwei Wang, Li Xia, Rong Li, Qiumin Zhang, Songqing Pan

**Affiliations:** ^1^Department of Neurology, Renmin Hospital, Wuhan University, Wuhan, China; ^2^Department of Clinical Laboratory, Renmin Hospital of Wuhan University, Wuhan, China

**Keywords:** reflex epilepsy, toothbrushing, SYN1 mutation, x-linked inheritance, whole-exome sequencing

## Abstract

Toothbrushing epilepsy is a rare form of reflex epilepsy (RE) with sporadic incidence. To characterize the genetic profile of reflex epilepsy patients with tooth brushing-induced seizures in a Chinese family. Solo clinical whole-exome sequencing (WES) of the proband, a 37-year-old Chinese man, was performed to characterize the genetic etiology of toothbrushing epilepsy. Mutations in the maternal X-linked synapsin 1 (SYN1) identified in the proband and his family members were confirmed by Sanger sequencing. The pathogenicity of these mutations was determined using in silico analysis. The proband had four episodes of toothbrushing-induced seizures. The semiology included nausea, twitching of the right side of the mouth and face, followed by a generalized tonic-clonic seizure (GTCS). The proband's elder maternal uncle had three toothbrushing-induced epileptic seizures at the age of 26. The proband's younger maternal uncle had no history of epileptic seizures but had a learning disability and aggressive tendencies. We identified a deleterious nonsense mutation, c.1807C>T (p.Q603Ter), in exon 12 of the *SYN1* gene (NM_006950), which can result in a truncated *SYN1* phosphoprotein with altered flexibility and hydropathicity. This novel mutation has not been reported in the 1000G, EVS, ExAC, gnomAD, or HGMD databases. We identified a novel X-linked *SYN1* exon 12 mutant gene in a Chinese family with toothbrushing epilepsy. Our findings provide novel insights into the mechanism of this complex form of reflex epilepsy that could potentially be applied in disease diagnosis.

## Introduction

Epilepsy is a common neurological disease that can arise from genetic or environmental factors, or a combination thereof. Reflex epilepsies (REs) are characterized by the presence of reflex seizures which consistently induced by a specific trigger (1). Photosensitive reflex-induced seizures are the most common type of reflex seizures. Other triggers that have been reported include music, reading, praxis, eating, hot water, and bathing (1).

Toothbrushing epilepsy is a rare and intriguing form of RE that can be triggered by using a manual or electric toothbrush (2), by active or passive toothbrushing (3), or even by the mere thought or sight of a toothbrush or toothpaste (4). Notably, toothbrushing-induced epileptic seizures appear to be rare, with 17 reported cases to date (2–13). Moreover, most toothbrushing epilepsy patients also suffer seizures unrelated to toothbrushing (4), including unprovoked seizures (3–5) as well as seizures due to oral-related triggers such as eating (11).

Although focal brain lesions, tumor, atrophy, cortical dysplasia, and gangliocytoma have been found in patients with toothbrushing epilepsy (6, 9, 13), brain lesions are not detected in the majority of patients. In addition, the semiology of seizures, neurological status, electroencephalogram (EEG), and brain magnetic resonance imaging (MRI) findings, and type and success of therapeutic intervention are highly heterogeneous across reports, adding to the complexity of this disease.

Given that the genetic etiology and underlying molecular mechanism of toothbrushing epilepsy and its progression toward unprovoked and difficult-to-treat seizures remain poorly understood, we conducted a solo whole-exome sequencing (WES) of a 37-year-old Chinese male proband to identify disease-related mutations. We next conducted Sanger sequencing and in silico analysis of the proband and all his family members. Our findings could have potential applications in improving the diagnostic accuracy of toothbrushing epilepsy.

## Methods

### Study Subjects

A 37-year-old Chinese man with reflex epilepsy admitted at the Renmin Hospital of Wuhan University, China was selected and recruited for this study. He was of normal intellect and experienced four episodes of toothbrushing-induced seizures within a period of 8 months. The proband's family members and extended family members were also enrolled in this study to trace the inheritance pattern of this type of RE. Written informed consent for genetic sequencing analysis and publication of personal photographs was obtained for all nine study participants. This study was approved by the Medical Ethics Committee of the Renmin Hospital of Wuhan University, China. All procedures were carried out in accordance with the ethical guidelines for human subject research.

### Whole Exome Sequencing (WES)

Solo clinical whole-exome sequencing (WES) of the proband was performed to identify the genetic etiology. Genomic DNA was extracted for library preparation using the Ion PI Ampliseq Exome RDY kit 18 × configuration (Applied Biosystems, Thermo Fisher Scientific Inc., Waltham, MA, USA). The Ion PI Hi-Q OT2 200 kit was used for template preparation. The amplicon libraries and template were prepared according to the manufacturer's instructions. Sequencing was conducted using a Proton Semiconductor Sequencer (Life Technologies, Thermo Fisher Scientific Inc.).

### Bioinformatics Analysis

Adapter sequences were trimmed from the raw sequence data. The processed data was then aligned to the hg19 human reference genome (GRCh37). Subsequently, interpretations of genomic variants were facilitated using the Ion Reporter software 5.12.

All identified variants and genomic regions below 20× coverage were visually verified using the Integrative Genomics Viewer (IGV) v2.3.8 (Broad Institute). Variants of genes related to inherited epilepsy syndrome were filtered and ranked according to presumed familial inheritance patterns.

### In *Silico* Analyses

In *silico* analyses were performed to determine the pathogenicity of the mutations that were identified. The identified alleles were compared with data reported in different allele frequencies from various human genetic variation databases, including gnomAD, ExAC, ESP, 1000G and Kaviar to exclude genetic variants previously reported as polymorphisms. We employed VarCards to interpret the missense variants with 23 in *silico* algorithms or tools (14–19).

The generation of 3D models and structure prediction of the wild-type and mutant forms of *SYN1* were performed using the Phyre2 (20) and I-TASSER software (21), respectively.

### Mutational Analysis and Sanger Sequencing

Sanger sequencing was performed on the proband and all his family members. The primers for the region of exon 12 of *SYN1* (NM_006950), including exon/intron junctions, were designed using Primer-BLAST (22), and synthesized by Invitrogen (Shanghai, China). The *SYN1* exon 12 primer sequences are as follows: for: 5′-ACAGGCTACCCGTCAGACAT-3′; and rev: 5′-TGGAGAGAGTTCGTGGGACC-3′. Polymerase chain reaction (PCR) was performed using a Veriti thermo cycler (Applied Biosystems, Foster City, CA, USA) at an annealing temperature of 60°C for 35 PCR cycles. The PCR product yield a band of 428 base pairs (bp). The amplified products were sequenced using an ABI 3,500 DNA sequencer (Applied Biosystems).

## Results

### Pedigree Analysis and Clinicopathology of the Proband and His Family

**Figure 1:**
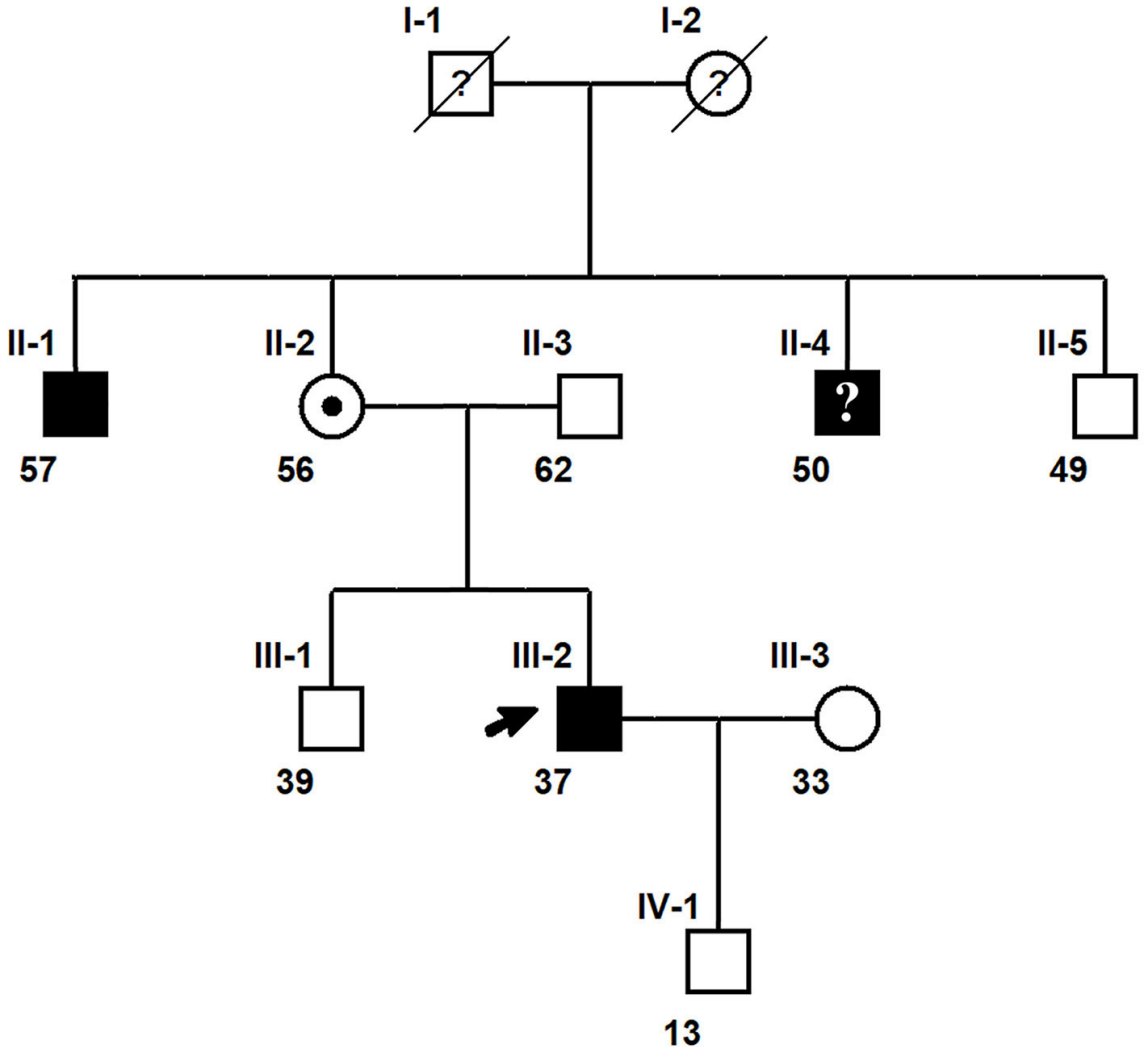
A three-generation pedigree chart of the proband with the *SYN1* mutation. Squares represent males, and circles denote females. Individuals with the *SYN1* mutation are represented with a shaded symbol while unaffected individuals are denoted with unshaded symbols. Roman numerals I, II, and II above each node indicate the generation while the numbers denote the number of individuals in each generation. The numbers at the bottom left of each node indicate the age of each individual. Individuals with unknown clinical diagnosis or uncertain diagnosis are denoted by a question mark (?) within the symbol. A slash through a symbol represents a deceased individual. The proband with the *SYN1* hemizygous c.1807C>T (p.Q603Ter) mutation is indicated with a black arrow. Individual II-4 has the *SYN1* mutation but did not experience RE. The proband's mother (circle with a dot) is a carrier of the mutant *SYN1* allele.

The inheritance model of the *SYN1* genetic variant was established by pedigree analysis of the 37-year-old Chinese male proband and his eight family members and extended family members (Figure 1). All affected members of the pedigree tree were male, indicating X-linked inheritance.

The semiology of toothbrushing-induced seizures experienced by the proband (Figure 1, Individual III:2) started a few seconds after toothbrushing onset, with nausea and twitching on the right side of the mouth and face, followed by loss of consciousness and a generalized tonic-clonic seizure (GTCS). The seizures could be induced by brushing the upper or lower teeth on either side of the mouth with the use of either hand. The proband knew the story about his uncle very well who also has toothbrushing induced seizures several years ago, so he stopped brushing his teeth for 1 month after experienced three episodes. However, when he resumed brushing teeth, the seizure recurred. After experienced totally four toothbrushing induced seizures, he stopped brushing his teeth and seizure free for around 6 months until he had another spontaneous GTCS during sleep. Oxcarbazepine 300 mg twice a day was then given and the seizures didn't recur for 1 year follow-up.

The proband had normal developmental history without any behavioral disorder. His MRI neuroimaging was normal, but his interictal EEG showed intermittent rhythmic slow waves in the left frontotemporal region. The patient underwent a long-term video-EEG for 36 h but toothbrushing seizures could not be induced despite he was four times asked to brushed his teeth.

The proband's 57-year-old elder maternal uncle (Figure 1, Individual II:1) had a history of three toothbrushing-induced epileptic seizures at the age of 26. The three occurrences of toothbrushing-induced seizures were initiated when he was brushing his tongue during toothbrushing. The subject experienced nausea, stiffness of the tongue, and twitching of the right side of his face before he fell down and lost consciousness. He recovered after around 3 min for all three occurrences. Following the third seizure episode, he stopped brushing his teeth and was seizure-free for 2 months. He then resumed brushing his teeth, taking care to avoid touching his tongue, and did not experience recurrence thereafter. He didn't experience any spontaneous seizure. He was of normal intelligence but experienced mild learning difficulties during childhood.

Although the proband's 50-year-old younger maternal uncle (Figure 1, Individual II:4) did not have a history of RE, he exhibited aggressive behaviors and experienced difficulties in learning. He did not complete primary school education and was unemployed.

### Proband Has a Nonsense Mutation in Exon 12 of the SYN1 Gene

Solo clinical WES of the proband led to the identification of a novel hemizygous *SYN1* variant c.1807C>T (p.Q603Ter). This variant arose due to a nonsense mutation in exon 12 of the *SYN1* gene on the chr23:47433576 G>A locus, causing the conversion to the amino acid glutamine at position 603 (Q603).

### The SYN1 Mutation Is Confirmed by Sanger Sequencing

**Figure 2:**
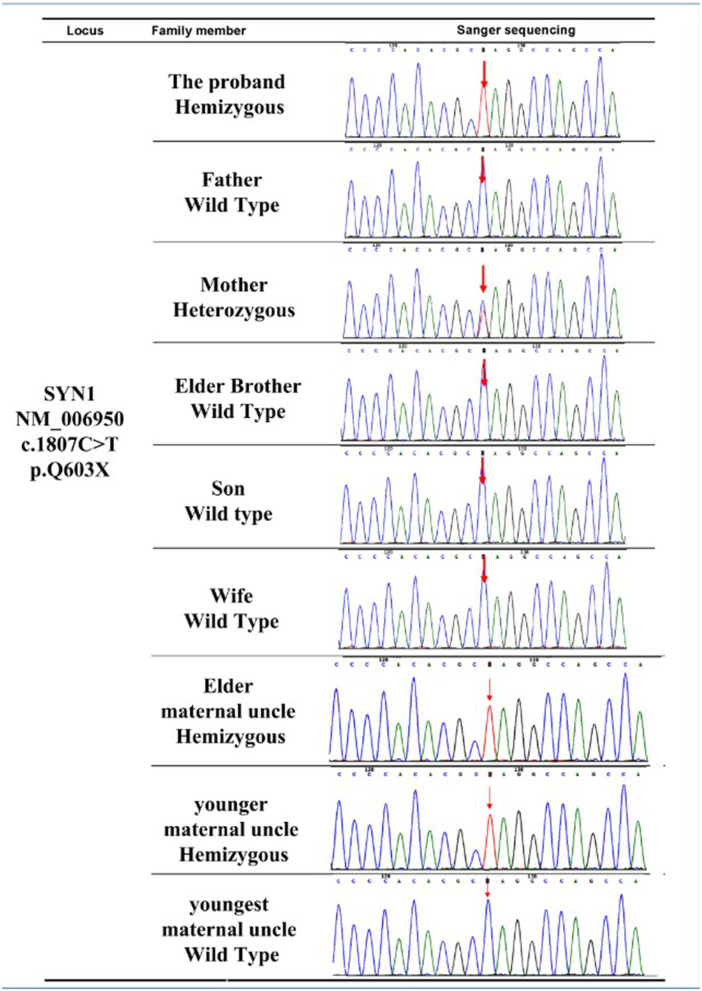
Multiple sequence alignment analysis of the *SYN1* mutation site. The mutant site (amino acid 603) is denoted by the red arrow.

Sanger sequencing (23) of the *SYN1* c.1807C>T (p.Q603Ter) mutation was performed on all family members except the proband's deceased maternal grandfather and deceased maternal grandmother. The Sanger sequencing results (Figure 2) confirmed the solo WES results for the proband and revealed that the proband's mother (Figure 1, Individual II-2) was a carrier of the *SYN1* genetic variant with a heterozygous genotype. Both the proband's affected elder maternal uncle (Figure 1, Individual II-1) and younger maternal uncle (Figure 1, Individual II-4) were X-linked hemizygotes for the *SYN1* mutant variant. The other five family members did not possess the mutant allele and did not have RE.

### The SYN1 Mutation Is Pathogenic and Highly Conserved

In silico algorithms or tools unanimously predicted the *SYN1* mutant protein to be 'disease-causing' and 'probably damaging', supporting the sequencing results and the clinicopathology observed (Supplementary Table 1). In addition, the *SYN1* mutant gene was found to be highly conserved across different species (Supplementary Figure 1 and Supplementary Table 2).

### The SYN1 Mutant Gene Encodes a Truncated Protein

The *SYN1* mutant gene was predicted by the I-TASSER software to encode a truncated *SYN1* protein lacking the C-terminus (Supplementary Figure 2). Absence of the C-terminus could compromise structural flexibility. The Grand average of hydropathicity (GRAVY) index was −0.628 for the wild-type, and 0.581 for the p.Q603Ter mutant, indicating that the *SYN1* mutant protein is more hydrophobic than the wild-type.

## Discussion

Multimodal investigations of brain function suggest that anatomical networks involved in modulating highly complex inherited or tightly-regulated physiological functions in humans may underlie RE pathogenesis (24).

Mutation (Q555X) of the X-linked *SYN1* gene that encodes a neuronal phosphoprotein synapsin I has been reported in patients with inherited reflex seizures that can be triggered by bathing, nail clipping, and face-rubbing, with potential epileptogenic network involving the temporo-insular region (25). Mutation of this particular gene (*SYN1*), albeit a different type of mutation, was identified in this study and was found to be correlated with toothbrushing epilepsy. It was recognized that shadow brushing was not able to induce the seizure (6), thus toothbrushing was deemed as a somatosensory rather than movement stimulus. Interestingly, oral and pharyngeal somatosensory inputs have been shown to have bilateral projections to the insular and frontal opercular cortex (26), which is close to the temporal-insular network associated with bathing induced reflex seizure.

Of note, the *SYN1* c.1807C>T (p.Q603Ter) nonsense mutation has not been reported in the literature (27–29), and there were no records of it in the 1000G, EVS, ExAC, gnomAD, and HGMD databases prior to this study.

Considering the role of the *SYN1* protein in modulating synaptic homeostasis, neurotransmission, neuronal development, synaptogenesis, maintenance of mature synapses, and neuronal plasticity (30), its implication in toothbrushing epilepsy was not surprising.

*SYN1* gene mutation can result in either a nonfunctional truncated protein or degradation of the messenger RNA (mRNA) before it can be translated. All three affected men in the family in this study were hemizygous for the *SYN1* c.1807C>T (p.Q603Ter) nonsense mutation, suggesting an association between the *SYN1* mutant allele and toothbrushing epilepsy.

Additionally, *SYN1* mutation is correlated with variable learning disabilities and behavioral disorders (OMIM #300491) (30, 31). The semiology for these three affected men lacking a functional *SYN1* protein was varied. Unlike the proband and his elder maternal uncle, the proband's younger maternal uncle had no history of toothbrushing epilepsy. However, he had learning disabilities and exhibited aggressive behavior. The relationship between the clinical phenotype and genetic etiology of epilepsy is complex and poorly understood. There are numerous forms of *SYN1*-related epilepsy including X-linked epilepsy with variable learning disabilities and behavior disorders, bathing epilepsy, hot water epilepsy that may involve GTCS and/or focal unaware seizures, with no fixed age of seizure onset or termination (32). The variable phenotypes observed among the affected family members may be attributed to environmental influences.

We employed the WES technique in the present study as it is a rapid and powerful approach for characterizing the genetic etiology of many diseases. A limitation of this study is the lack of functional evaluation, which we were unable to perform due to technical limitations. Nonetheless, we aim to perform these experiments in future follow-up studies if circumstances permit.

## Conclusion

Toothbrushing epilepsy is regarded as a rare disorder with poorly characterized genetic etiology. Here, we found an association between toothbrushing epilepsy and *SYN1* gene mutation, and added toothbrushing as another environmental trigger for *SYN1*-related RE. Our study provides novel insights into the genetic etiology of toothbrushing epilepsy. Furthermore, our analyses suggested the potential of *SYN1* nonsense mutation as a candidate biomarker for the diagnosis of toothbrushing epilepsy. Genetic counseling and early prenatal diagnosis of patients with a family history of toothbrushing epilepsy could potentially facilitate family planning decision-making.

## Data Availability Statement

The data presented in the study are deposited in the SRA database repository (https://www.ncbi.nlm.nih.gov/sra/PRJNA751964), accession number PRJNA751964.

## Ethics Statement

The studies involving human participants were reviewed and approved by Medical Ethics Committee of the Renmin Hospital of Wuhan University, China. Written informed consent to participate in this study was provided by the participants' legal guardian/next of kin.

## Author Contributions

QinZ: design the study, collect clinical and genetic data, and write manuscript. JW: genetic analysis, and write the genetic section of the manuscript. LX: collect clinical and EEG data. RL: collect clinical data, and communicate with patients and family members. QiuZ: collect clinical data. SP: design the study, formulate study hypothesis, and communicate with patients and family members. All authors contributed to the article and approved the submitted version.

## Conflict of Interest

The authors declare that the research was conducted in the absence of any commercial or financial relationships that could be construed as a potential conflict of interest.

## Publisher's Note

All claims expressed in this article are solely those of the authors and do not necessarily represent those of their affiliated organizations, or those of the publisher, the editors and the reviewers. Any product that may be evaluated in this article, or claim that may be made by its manufacturer, is not guaranteed or endorsed by the publisher.
