# Supplementary figures and images for: SYN1 Mutation Causes X-Linked Toothbrushing Epilepsy in a Chinese Family
Source: Front Neurol. 2021 Sep 20;12:736977. doi: 10.3389/fneur.2021.736977 (PMC8488375; doi:10.3389/fneur.2021.736977)

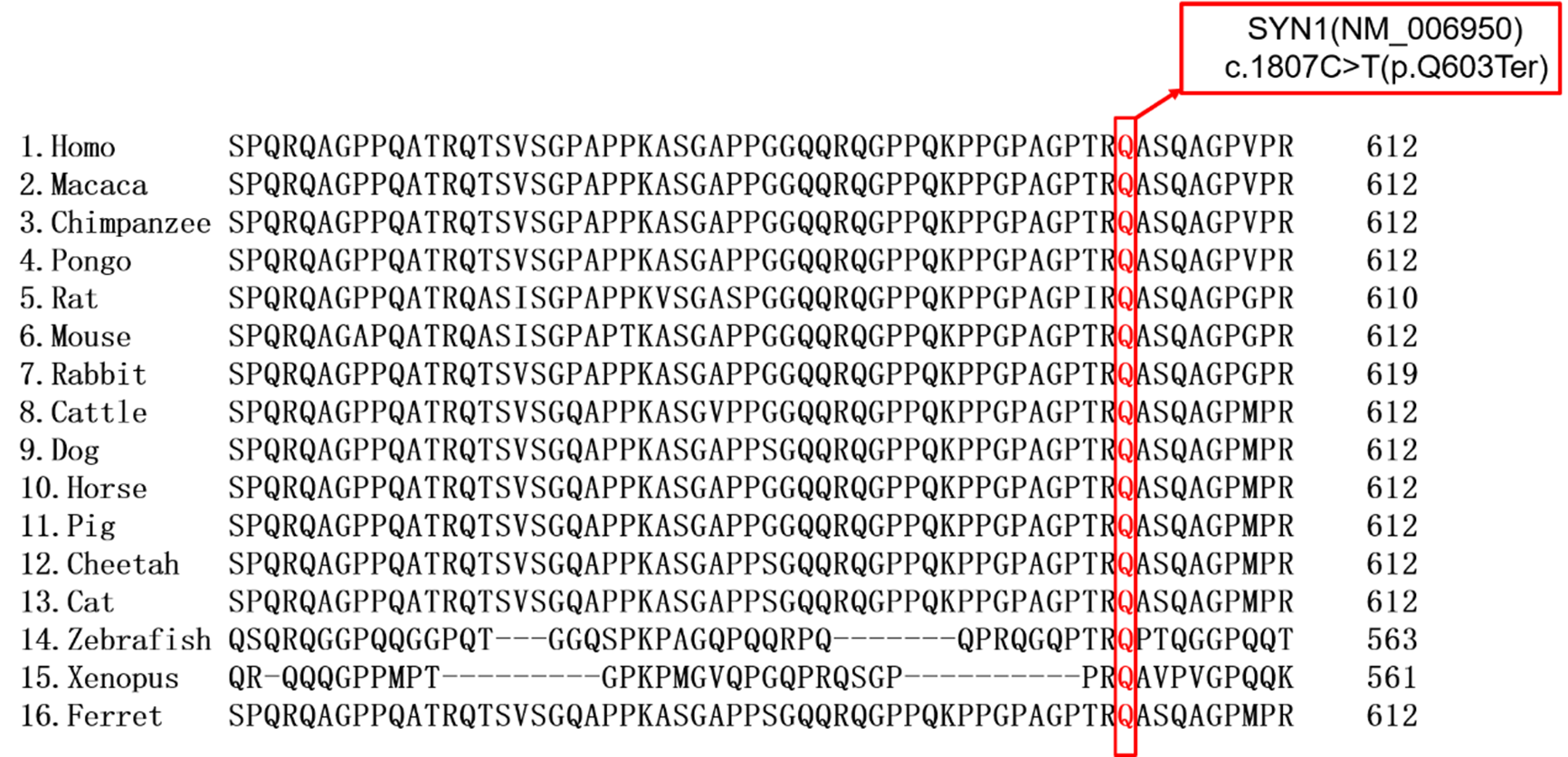

Supplement: Supplementary file 1 [file Image_1.png]

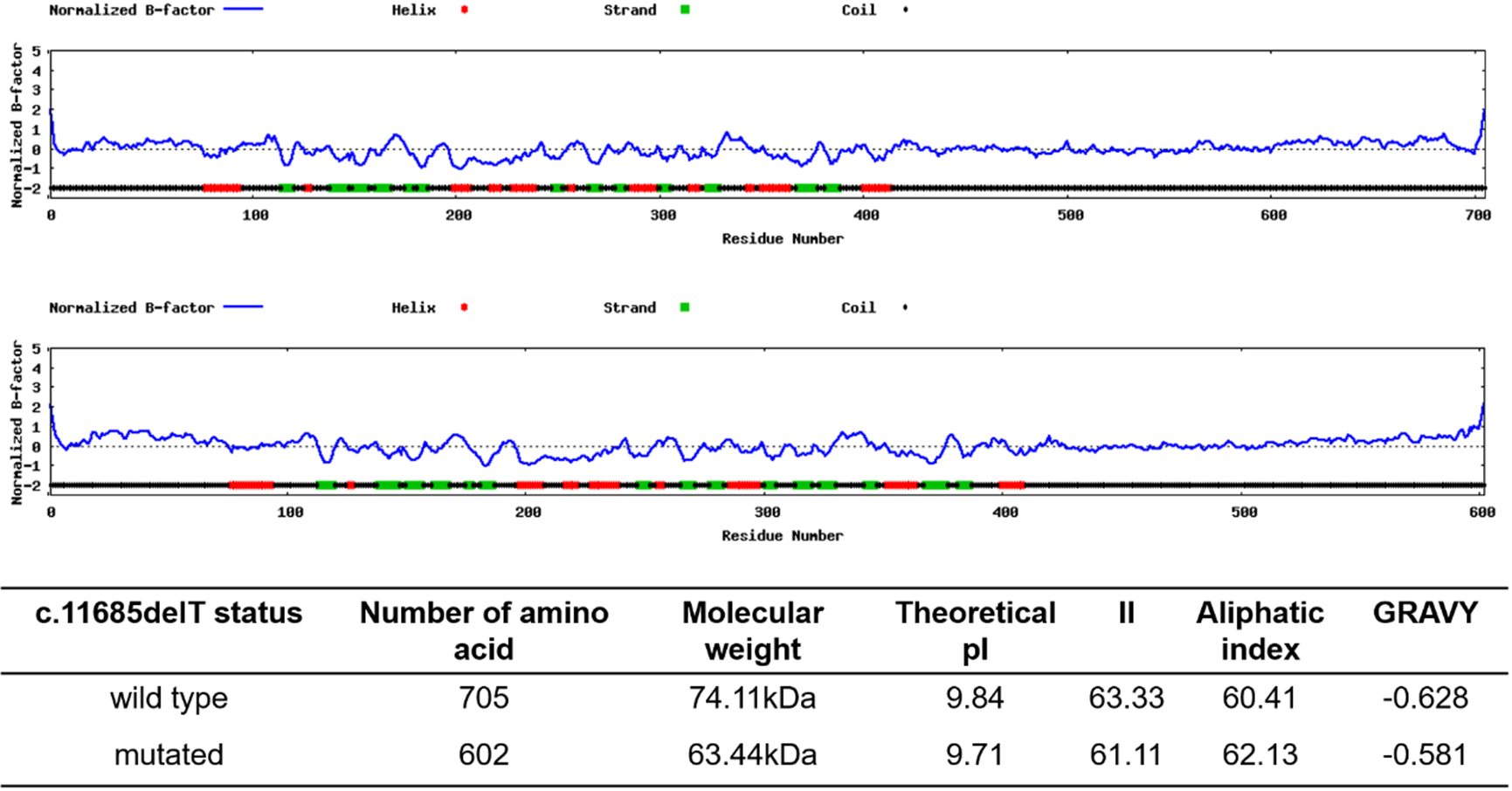

Supplement: Supplementary file 2 [file Image_2.png]
