# Supplementary material for: SYN1 Mutation Causes X-Linked Toothbrushing Epilepsy in a Chinese Family
Source: Front Neurol. 2021 Sep 20;12:736977. doi: 10.3389/fneur.2021.736977 (PMC8488375; doi:10.3389/fneur.2021.736977)
Supplement: Supplementary file 3 [file Table_1.DOCX]

**Supplemental files**

**Table 1. Phenotypic/functional effect of the *SYN1* nonsense mutation.**

| Software  Mutation | Damaging Score (phenotypical/functional effect prediction) | | | | |
| --- | --- | --- | --- | --- | --- |
|  | MutationTaster | GenoCanyon | FATHMM_MKL | CADD | DANN |
| p.Q603Ter | 1 (Disease_causing) | 1 (Damaging) | 0.901 (Damaging) | 39 (Damaging) | 0.997 (Damaging) |

Note: Score indicates the raw score for the corresponding function prediction/conservation score output. The deleterious threshold was used as a filtering criterion for qualitative prediction score analysis to classify the possible outcomes of the *SYN1* genetic mutation. MutationTaster assigns a score between 0 and 1, with a deleterious threshold > 0.5. GenoCanyon has a scoring system between 0 and 1, with a cutoff at > 0.5. Both DANN and FATHMM assign scores between 0 and 1, with a deleterious threshold of > 0.96 and > 0.80, respectively. CADD has an open-ended scoring system ranging from around -7 to 20, with a deleterious threshold > 1.75.

**Table 2. Evolutionary conservation of the *SYN1* mutation.**

| Software  Mutation | Conservation score (evolutionary conservation prediction) | | | |
| --- | --- | --- | --- | --- |
|  | GERP | phyloP | phastCons | SiPhy |
| p.Q603Ter | 4.52 (Conserved) | 3.058 (Conserved) | 1 (Conserved) | 12.08 (Conserved) |

Note: Score indicates raw score for the corresponding conservation score output. GERP assigns scores between -15 and 10. PhyloP assigns scores between 3 and 14, with a threshold > 4.4. PhastCon assigns scores between 0 and 1. SiPhy assigns scores between 0 and 20, with a cutoff > 12.17. Higher scores indicate a higher likelihood the *SYN1* mutation is evolutionarily conserved.
